# Myoclone Assay: Clonogenic Expansion of Activating Muscle Stem Cells

**DOI:** 10.21769/BioProtoc.5869

**Published:** 2026-11-05

**Authors:** M. Pilar J. Stella, Oscar Damgaard, Zofija Frimand, Antoine De Morrée

**Affiliations:** Department of Biomedicine, Aarhus University, Aarhus, Denmark

**Keywords:** Clonogenic expansion, Muscle stem cells, Activation, Proliferation, Quiescence

## Abstract

Muscle regeneration depends on the function of adult muscle stem cells (MuSCs). During homeostasis, MuSCs reside in a quiescent state of prolonged reversible cell cycle exit. Upon muscle injury, MuSCs exit the quiescent state and activate to enter the cell cycle. Subsequently, they undergo rounds of clonogenic expansion before differentiating to form new muscle fibers. Any impairment to the MuSCs’ ability to switch between these cell states can impair muscle regeneration. To study MuSC function, it is therefore important to quantitatively assess MuSCs’ capacity to switch between cell states. Numerous robust methods have been developed that assess the activation and proliferation capacity of MuSCs. However, these methods often require thousands of cells, and MuSCs are rare. Here, we present a protocol to assess MuSC activation using only 60 cells per sample by monitoring the parallel clonogenic expansion of single MuSCs. Single primary MuSCs are seeded into 96-well half-area microplates at a 1-cell-per-well density, which takes as little as 2 min per sample. The cells are then incubated for 8 days in the presence of growth factors, stained with Hoechst, and imaged by inverse fluorescence microscopy. We demonstrate that relative clonogenic expansion data provide results that are similar to published data obtained using other methods, such as cumulative EdU incorporation assays. This myoclone protocol enables the quantitative assessment of MuSC activation using only 60 cells, in a time-efficient and cost-effective manner, and thereby can provide new insights into MuSC function.

Key features

• Requires a minimum of 60 cells to assess clonogenic expansion of a MuSC population.

• Can easily be included in any workflow that involves FACS.

• Uses an easy-to-score phenotype of clonogenic expansion that does not require specific software.

• Enables downstream assays with clonally expanded cell cultures.

## Graphical overview


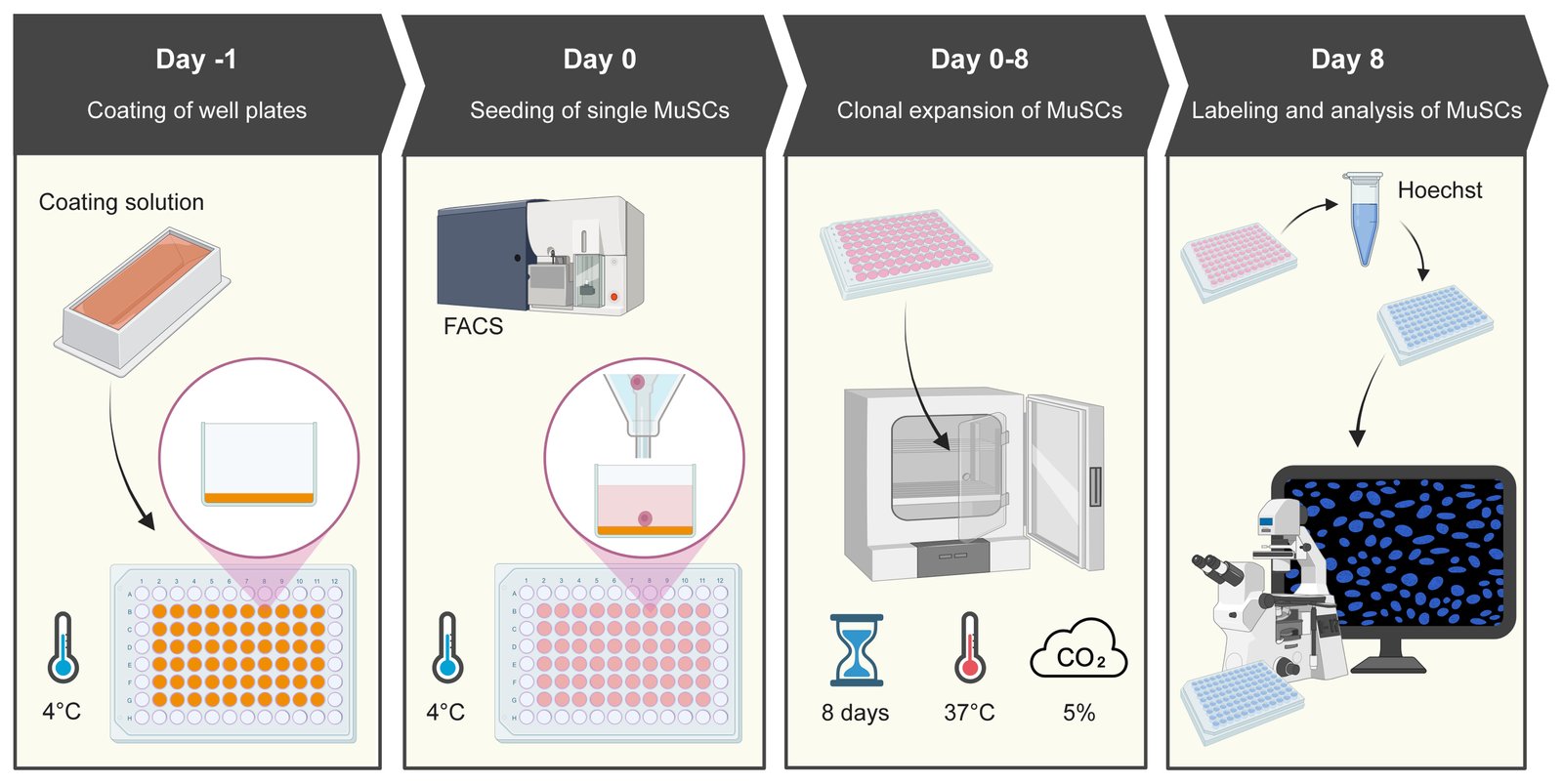


**Procedure schematics.** The schematic shows the four main procedures of the protocol.

## Background

Adult stem cells are paramount for tissue regeneration, given their ability to rapidly expand into large numbers of differentiation-competent cells that can rebuild tissue [1,2]. One well-studied adult stem cell model is the muscle stem cell (MuSC). MuSCs preserve skeletal muscle function and drive its regeneration. MuSCs are rare cells that reside in a quiescent state of prolonged cell cycle exit. Upon injury, MuSCs activate to exit the quiescent state, proliferate, and differentiate to make new myofibers [2–4]. Some MuSCs self-renew and re-enter the quiescent state to repopulate the niche in anticipation of future muscle damage [5].

Understanding MuSC function requires assays to quantitatively assess the processes of activation and proliferation. Nucleotide analogs are the gold standard for assessing MuSC activation and proliferation. However, such experiments require thousands of cells, and MuSCs are rare [6,7]. In other adult stem cell models, the ability of stem cells to activate and replicate into undifferentiated daughter cells is commonly tested using clonogenic expansion assays [8]. These assays use low numbers of single cells as input and can reveal altered stem cell activation, tissue function, and impacted outcomes of tissue regeneration following transplants [9,10]. Here, we present the myoclone assay, a protocol for the quantitative assessment of MuSC activation and clonogenic growth based on our recent publication [11].

The myoclone assay requires 60 cells per sample, a much lower number than in other assays that require thousands of cells per sample [7,12–14]. MuSCs are commonly isolated from skeletal muscles using fluorescence-activated cell sorting (FACS) [7,15–19]. Given the low cell number required, the myoclone assay can easily be added to any experimental plan involving FACS. Moreover, the low cell number greatly cuts the required sort time, which results in cost savings and the ability to analyze more samples in parallel. Finally, since the MuSCs remain alive in the microwells at the end of the assay, the myoclone assay can be combined with downstream protocols such as cell culture and immunostaining.

Here, we demonstrate the sensitivity of the myoclone assay using MuSCs isolated from diaphragm and hindlimb muscles of young mice. MuSCs isolated from these two muscle groups are known to have different activation and proliferation dynamics [6].

## Materials and reagents

**Biological materials**

1. Primary muscle stem cells (MuSCs) (e.g., isolated from *Mus musculus* in-house)

**Reagents**

1. Laminin mouse protein, natural (Gibco, catalog number: 23017015)

2. Bovine collagen type 1 (Sigma-Aldrich, catalog number: C8919)

3. Anhydrous acetic acid (Merck, catalog number: 1.00063.1000)

4. PBS tablets (Gibco, catalog number: 18912014)

5. Ham’s F-10 nutrient mix (Gibco, catalog number: 11574436)

6. Horse serum heat-inactivated, New Zealand origin (Thermo Fisher, catalog number: 26050088)

7. Penicillin-streptomycin (Gibco, catalog number: 15140122)

8. Human FGF-basic (FGF-2/bFGF) (154 aa) recombinant protein, PeproTech (Gibco, catalog number: 100-18B)

9. Hoechst 33342 (Thermo Scientific, catalog number: 62249)

10. Biotin anti-mouse CD106 antibody, clone 429 (BioLegend, catalog number: 105704)

11. PE/Cyanine7 Streptavidin (BioLegend, catalog number: 405206)

12. FITC anti-mouse CD45 antibody, clone 30-F11 (BioLegend, catalog number: 103108)

13. FITC anti-mouse CD31 antibody, clone MEC13.3 (BioLegend, catalog number: 102506)

14. Pacific Blue anti-mouse Ly-6A/E (Sca-1) antibody, clone D7 (BioLegend, catalog number: 108120)

**Solutions**

1. Basal myoclone medium (see Recipes)

2. 1× PBS (see Recipes)

3. Laminin stock solution (see Recipes)

4. Collagen stock solution (see Recipes)

**Recipes**

**1. Basal myoclone medium**

**Table:** 

Reagent	Final concentration	Quantity or volume
Ham’s F-10 nutrient mix	NA	500 mL
Horse serum heat-inactivated	20%	100 mL
Penicillin-streptomycin	1% Penicillin (100 units/mL) 1% Streptomycin (0.1 mg/mL)	5 mL

Thaw 100 mL of horse serum (HS) and 5 mL of penicillin-streptomycin (Pen/Strep, 10,000 units/mL / 10 mg/mL) at 37 °C in a water bath. Once thawed, bring the HS and Pen/Strep into a laminar flow hood (LAF) together with an unopened bottle of Ham’s F-10 (500 mL). Add 100 mL of HS and 5 mL of Pen/Strep to the 500 mL bottle of Ham’s F-10 after taking out and discarding 105 mL of the F-10. Mix the medium by gently inverting the bottle to avoid the formation of bubbles. Store the bottle of basal myoclone medium at 4 °C.

**2. 1× PBS**

**Table:** 

Reagent	Final concentration	Quantity or volume
PBS tablets	1× (10 g/L)	5 g
Milli-Q water	NA	500 mL

Open the vial of PBS tablets in the LAF. Dissolve one 5 g sterile PBS tablet in 500 mL of Milli-Q water in a 500 mL borosilicate glass bottle. Mix using a stir bar and a magnetic rotator. Alternatively, leave the PBS tablet to dissolve in the Milli-Q water for 1 h and swirl to mix afterward. Filter the PBS using a 0.45-μm, 500-mL polyethersulfone filter unit attached to an autoclaved 500 mL borosilicate bottle. Store the sterile PBS at 4 °C.

**3. Laminin stock solution**

**Table:** 

Reagent	Final concentration	Quantity or volume
Laminin mouse protein	0.02 mg/mL	1 mg
Sterile 1× PBS	NA	50 mL

Retrieve Laminin mouse protein from the -80 °C freezer and thaw. Once thawed, keep on ice until use to prevent gel formation. Bring filter-sterilized 1× PBS and the laminin to the LAF. Transfer the total 1 mL volume of Laminin mouse protein (1 mg/mL) from its vial into a 50 mL borosilicate glass bottle with 49 mL of sterile 1× PBS to obtain a laminin stock solution at a final concentration of 0.02 mg/mL. Swirl to mix. Aliquot as desired (e.g., 5 mL aliquots) and store at -80 °C until use. Note that the vendor can provide Laminin mouse protein at varying concentrations, and the volumes may need adjustment to obtain a final concentration of 0.02 mg/mL.

**4. Collagen stock solution**

**Table:** 

Reagent	Final concentration	Quantity or volume
Anhydrous acetic acid	100 mM	5.15 mL
Milli-Q water	NA	895 mL

**Caution:** This step involves working with highly concentrated acid. Make sure to wear personal protective equipment (lab coat, gloves, closed-toe shoes, protective eyewear) and work in a chemical fume hood.

Collect filtered Milli-Q water from a Milli-Q water dispenser. Bring anhydrous acetic acid and Milli-Q water to the fume hood. To prepare acidified water, transfer 895 mL of sterile Milli-Q water to a borosilicate glass bottle and add 5.15 mL of anhydrous acetic acid to the Milli-Q water.

**Table:** 

Reagent	Final concentration	Quantity or volume
Bovine collagen type 1	0.05 mg/mL	10 mL (1 mg/mL)
Acidified water	95 mM in collagen stock solution	190 mL

Transfer 190 mL of acidified water to a 200 mL borosilicate glass bottle. Add 10 mL of bovine collagen type 1 (10 mg) to the 200 mL borosilicate glass bottle to obtain the final collagen stock solution (0.05 mg/mL). Swirl to mix. Filter the collagen stock solution using a 0.45-μm, 500-mL polyethersulfone filter unit attached to an autoclaved 200 mL borosilicate bottle. After filtration, swirl the solution to mix. Store the collagen stock solution at 4 °C until use.

**Laboratory supplies**

1. Cell culture plates, 96-well, CELLSTAR, half area, black, with lid (VWR, catalog number: 392-0058)

2. VWR^®^ LTS^®^ compatible filter tips, 1,000 μL micropipette tips (VWR, catalog number: 732-3642)

3. VWR^®^ LTS^®^ compatible filter tips, 250 μL micropipette tips (VWR, catalog number: 613-6503)

4. 25 mL serological pipette tips (Hounisen, catalog number: 86.1685.001)

5. 15 mL conical tubes, 120 × 17 mm, PP DNA-, DNAse-, RNase-free, non-pyrogenic, non-cytotoxic (Hounisen, catalog number: 62.554.502)

6. 50 mL conical tubes, 114 × 28 mm, PP DNA-, DNAse-, RNase-free, non-pyrogenic, non-cytotoxic (Hounisen, catalog number: 62.547.254)

7. DURAN^®^ BlueCap bottle, 250 mL borosilicate glass bottle (Hounisen, catalog number: 0610.0250)

8. DURAN^®^ BlueCap bottle, 50 mL borosilicate glass bottle (Hounisen, catalog number: 0610.0050)

9. 50 mL reservoir for multichannel micropipette, sterile (Hounisen, catalog number: 0699.8002)

10. 0.45 μm polyethersulfone (PES) Filter Unit (Fisher Scientific, catalog number: 15923307)

## Equipment

1. BD FACSAria^TM^III Sorter (BD Biosciences, catalog number: 648282-24)

2. Widefield Inverted Light Microscope, AxioObserver (Zeiss) with objectives 10×/NA 0.30 Air, Ph1, EC Plan-Neofluar and 5×/NA0.16 Air, Ph1, EC Plan-Neofluar

3. Eppendorf centrifuge 5810 (Eppendorf, catalog number: EP022628188)

4. Fisherbrand^TM^ Mini Tube Rotator (Fisher Scientific, catalog number: 15534080)

5. Thermo Scientific^TM^ Series 8000 Direct-Heat CO_2_ Incubator, 184 L (Fisher Scientific, catalog number: 11862711)

6. LAF: NinoSAFE Class II Biological Safety Cabinet (Nino Labinterior, catalog number: 101861)

7. Corning^®^ Stripettor^TM^ Ultra Pipette Controller (Corning, catalog number: 4099)

8. RAININ Pipet-Lite LTS Pipette L-20XLS+ (Mettler Toledo, catalog number: 17014392)

9. RAININ Pipet-Lite LTS Pipette L-200XLS+ (Mettler Toledo, catalog number: 17014391)

10. RAININ Pipet-Lite LTS Pipette L-1000XLS+ (Mettler Toledo, catalog number: 17014382)

11. RAININ Pipet-Lite Multi Pipette L8-200XLS+ (Mettler Toledo, catalog number: 17013805)

12. Graduated cylinder

13. Ice machine

14. Fume hood

15. Water bath (Thermo Fisher Scientific, catalog number: FSSWB15)

16. Ice bucket

17. Freezer (-20 °C)

18. Refrigerator (2–8 °C)

19. Autoclave

20. Arium Pro DI Ultrapure Water System (Sartorius, catalog number: H2OPRO-DI-T)

## Software and datasets

1. GraphPad Prism (GraphPad Software Inc., v.10.4.1)

2. Fiji image processing package (ImageJ2, v.1.54p)

## Procedure

**A. Coating microplates (day before cell sorting)**

1. Retrieve the required number of aliquots of laminin stock solution (0.02 mg/mL) from -80 °C and keep on ice to avoid gelation. Retrieve the collagen stock solution (0.05 mg/mL) from 4 °C. The protocol requires 1 mL of laminin stock solution and 1 mL of collagen stock solution per 96-well plate.

2. In the LAF, prepare a 1:1 laminin-collagen coating solution to a final concentration of 0.01 mg/mL laminin and 0.025 mg/mL collagen. For 8 plates, add 8 mL of laminin stock solution and 8 mL of collagen stock solution to a 50 mL borosilicate glass bottle. Swirl to mix.

3. Transfer the total volume of laminin-collagen coating solution (16 mL in the example) to a 50 mL reservoir. If working with large volumes of laminin-collagen coating solution, keep the borosilicate glass bottle with the coating solution on ice to prevent gelation. Add 30 μL/well of laminin-collagen coating solution to 60 wells of the 96-well plates, coating rows B–G and columns 2–11.

4. Spin the plates at 200× *g* for 1 min at 4 °C to remove air bubbles. Immediately transfer the plates to 4 °C to coat overnight.

**B. Cell isolation, sorting, and culture**

1. Preparation of a single-cell suspension that includes MuSCs

a. For example, sacrifice mice and isolate lower hindlimb and diaphragm muscles. Digest them into two single-cell suspensions and stain them with fluorescently labeled antibodies against cell surface markers, as, for example, described in Frimand et al. [7].

b. Start up the fluorescence-activated cell sorter and set up the gating strategy for single-cell sorting of mouse MuSCs, as, for example, described in Das Barman et al. [20].

2. Washing of microplates

a. Bring sterile 1× PBS, 500 mL of Ham’s F10 cell medium, 100 mL of horse serum, 5 mL of Pen/Strep, and two 50 mL reservoirs to the LAF. Prepare basal myoclone medium as described (see Recipes).

b. Retrieve the 96-well plates with the laminin-collagen coating solution from 4 °C. To discard the coating solution, remove the lid from the well plates, hold the well plate with one hand, and expel the liquid from the wells by performing a sharp downward motion in a sink while steadily holding the well plate.

c. In the LAF, wash the 96-well plates by adding 60 μL of sterile 1× PBS per well. Discard the wash in the sink with a sharp downward motion as described above. Repeat this wash three times.

d. To prepare final myoclone medium containing FGF at a concentration of 5 ng/mL, transfer 50 mL of basal myoclone medium into a 50 mL conical tube, retrieve 15 μL of human FGF-basic [FGF-2/bFGF (25 mg/mL)] from -20 °C, and add this to the 50 mL conical tube. Swirl to mix, transfer the final myoclone medium to a 50 mL reservoir, and pipette 100 μL into each of the coated wells. Store the plates at 4 °C until use.

3. MuSCs sorting and culture

a. Load one well plate at a time onto the plate stage (ACDU, or Automated Cell Deposition Unit) of the fluorescence-activated cell sorter. Sort one cell per well in each of the 60 wells of each coated well plate. As an optional but recommended control, sort 60 cells instead of 1 in the final of the 60 wells of each coated well plate. This control will help during microscopy to find the focal plane of the cells.

b. Incubate the well plates with the sorted MuSCs at 37 °C, 5% CO_2_, for 8 days without changing the medium. Periodically check the myoclone plates to ensure the myoclone medium has not evaporated. In case of evaporation, add additional myoclone medium.

**C. Analysis (8 days after cell sorting)**

1. Myoclone staining with Hoechst

**Caution:** Do not expel liquid from each well as described for washing microplates, as a vigorous downward motion could dislodge and expel cells from the well plates.

**Caution:** Hoechst is cytotoxic and stains both live and dying cells. Stain and perform microscopy of plates in small batches to avoid exposing the cells to Hoechst for longer periods of time, which could lead to cell death, thus impacting cell counting. Here, we stained four plates at a time and imaged them in sequence. See also General note 3.

a. Retrieve basal myoclone medium from 4 °C. Transfer the required amount to a 50 mL conical tube and warm up to 37 °C. Bring sterile 1× PBS, Hoechst, and three 50 mL reservoirs to the LAF.

b. Retrieve the 96-well plates from the incubator. Without disturbing the cells or touching the bottom of the wells, gently aspirate 90 μL of myoclone medium from each well of the 96-well plates using a pipette.

c. Prepare a 1:2,000 Hoechst dilution in warmed basal myoclone medium by adding the required amount of Hoechst to the 50 mL conical tube. Swirl to mix. Transfer the Hoechst staining solution in basal myoclone medium to a 50 mL reservoir and pipette 50 μL of the staining solution into each well of the 96-well plates. Incubate the plates in the incubator for 30 min.

d. After incubation, gently aspirate 50 μL of Hoechst staining solution from the wells of the 96-well plates and discard to a 50 mL waste reservoir.

e. Wash with sterile 1× PBS by pipetting 100 μL of 1× PBS into the designated wells of the 96-well plates. Next, discard the 1× PBS by gently aspirating 100 μL from each well and pipetting into a waste reservoir. Repeat this step three times.

f. Add 100 μL of basal myoclone medium to each of the wells. The plates are now ready for microscopy analysis.

2. Myoclone imaging

a. Set up the widefield inverted fluorescence microscope with a sample stage for plate imaging.

b. Select a nuclear counterstaining imaging channel to image the cells’ nuclei (DAPI or Hoechst, depending on the microscope settings).

c. Select the objective that provides the best trade-off between large field of view and cell detection and identification. Objectives with small magnification and small numerical aperture allow imaging large fields of view. They also allow detecting cells localized at different depths in the coating due to the wide depth of focus of the objective but can hinder cell identification. Objectives with large magnification and larger numerical aperture allow a more confident identification of cells. They also extend the time required for analysis due to smaller fields of view and smaller depth of focus. Here, we used a 5× objective (NA 0.16 Air) and a 10× objective (NA 0.30 Air).

d. Image the well of the well plate that was seeded with 60 cells. This well provides a control: the 60 cells should have proliferated into hundreds of cells by the end of the incubation period.

e. Browse through the wells of each of the 96-well plates by imaging with the nuclear counterstaining fluorescence channel. For each well, score a positive myoclone if the well contains at least 16 cells/well by manually counting the cells per well.

f. After imaging the 60 wells per well plate, return the well plates to the incubator for future experiments.

## Data analysis

To demonstrate the protocol, we seeded single MuSCs from diaphragm and lower hindlimb muscles of four wild-type C57/BL6 mice into freshly coated 96-well half-area microplates. Following cell culture, we stained the cells with Hoechst and scored the number of myoclones as a percentage of total wells. We used 16 cells per well as the cutoff to call myoclones. Using this criterion, we measured a significantly larger number of myoclones formed from the diaphragm MuSCs compared to lower hindlimb MuSCs (Figure 1), suggesting higher clonogenic capacity of diaphragm MuSCs. These results are similar to our results based on EdU incorporation, which showed a significantly higher propensity to activate in diaphragm MuSCs compared to lower hindlimb MuSCs [6,7].

**Figure 1.**
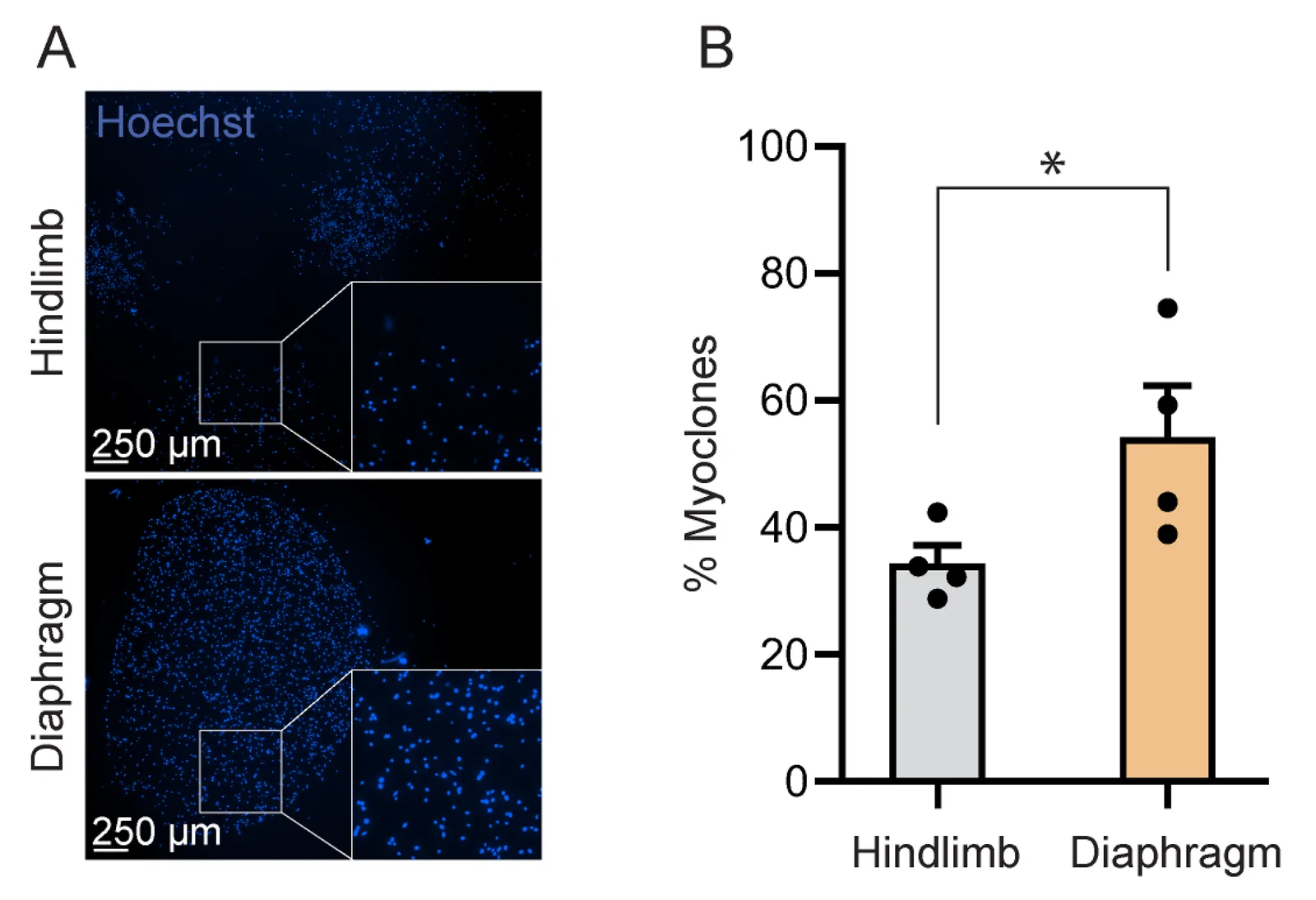
Quantification of clonogenic growth by number of formed myoclones. (A) Representative images of myoclones grown from single MuSCs. MuSCs from the mouse lower hindlimb (top panel) and diaphragm (bottom panel) are stained with Hoechst dye. Images were acquired at 10× magnification. Insets correspond to the areas noted by the boxes, magnified with ImageJ for visualization. (B) Bar graph showing the percentage of myoclones. Each dot represents a mouse. Bar graphs show mean ± SEM (n = 4). Statistical analysis was performed in GraphPad using a one-tailed unpaired t-test with Welch’s correction (*p < 0.05, p = 0.0422).

The lower clonogenic capacity in lower hindlimb MuSCs led us to ask whether some of the lower hindlimb MuSCs had simply not expanded yet to 16 cells. To this end, we plotted the data as a histogram of the clone sizes. We counted the number of cells per well for all wells in which we could detect fewer than 40 cells and binned them based on the number of cells per clone. We defined the bin ranges to account for the number of cell divisions completed during the 8 days (Figure 2). We observed a U-shaped pattern in the frequency of clone sizes for both lower hindlimb and diaphragm MuSCs. A similar percentage of lower hindlimb MuSCs and diaphragm MuSCs had no cells at all (approximately 33%), suggesting that the seeded cell had died during adhesion or the subsequent cell cycle. Lower hindlimb MuSCs had a significantly higher propensity to form a small clone (1–8 cells), yet a significantly lower propensity to form a large clone (>40 cells), compared to diaphragm MuSCs (Figure 2). There was no significant difference for clones of medium size (8–16 cells and 16–40 cells) (Figure 2).

**Figure 2.**
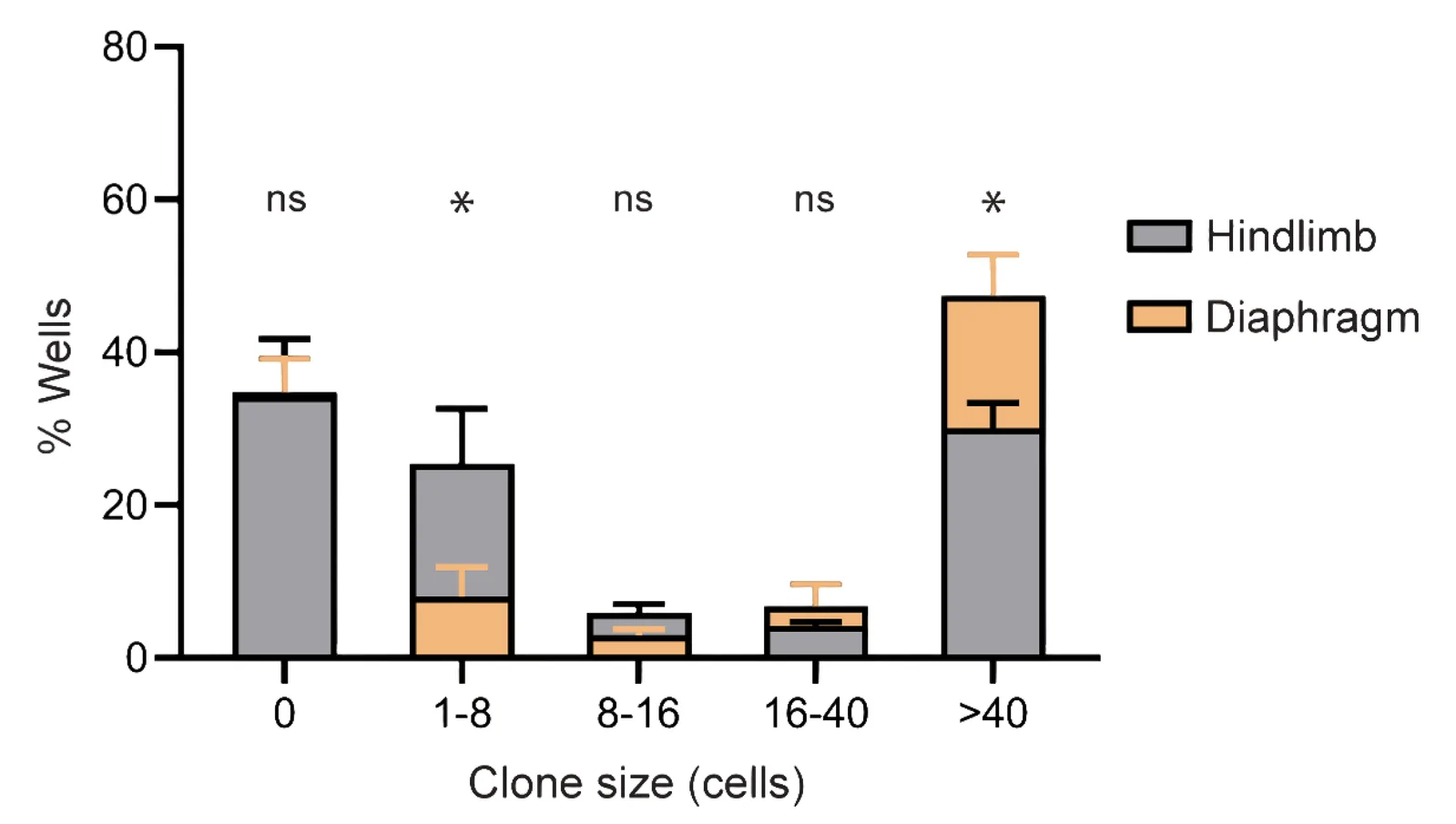
Classification of myoclones based on number of cells detected per well. Bar graph showing the percentage of cell clones formed grouped into bins of different clone sizes. Clones cultured from diaphragm MuSCs (orange bars) and from lower hindlimb MuSCs (gray bars) are superimposed. Bar graph shows mean ± SEM (n = 4). Statistical analysis was performed in GraphPad to compare the two tissues for each bin size separately using a one-tailed unpaired t-test with Welch’s correction (*p < 0.05, ns, non-significant).

## Validation of protocol

**This protocol has been used and validated in the following research article:**

• Kang et al. [11]. In vivo self-renewal and expansion of quiescent stem cells from a non-human primate. *Nature Communication*. https://doi.org/10.1038/s41467-025-58897-x

## General notes and troubleshooting

**General notes**

1. This protocol tests for the activation and proliferation potential of single MuSCs as a readout of MuSC activation that requires low cell input. Using this assay, we show that clonogenic capacity is higher in MuSCs isolated from the diaphragm, compared to their lower hindlimb counterparts (Figure 1), in accordance with our previous studies [6,7]. Skewed activation kinetics in the MuSCs from these muscle groups could result in the cells going through different numbers of cell divisions during the allotted time and may lead to diverse clonogenic expansion after 8 days of cell culture (Figures 1 and 2). In this sense, the clonogenic capacity of the MuSCs measured as the number of formed myoclones can be interpreted as a readout of stem cell activation. This assessment of clonogenic capacity and activation of MuSCs presented here, in accordance with previous cell population studies, reveals a higher clonogenic capacity of diaphragm MuSCs than hindlimb MuSCs, and can be interpreted as a more prompt activation of diaphragm MuSCs.

2. The protocol requires as few as 60 cells per sample and provides a straightforward binary readout of MuSC expansion potential and insights on MuSC activation. This is the main advantage of the assay. At the same time, the statistical variance computed from such a small sample size can be strongly impacted by protocol variations. The EdU incorporation assay mediates these characteristics, assessing activation and proliferation in cell populations. The EdU assay remains the gold standard for assessment of MuSC activation of cell populations [7,12,14]. However, it can be complemented by the myoclone assay.

3. Several experimental conditions are critical for the assay, which could result in considerable variability given the small number of cells used. The success of clonogenic expansion assays is strongly linked to the type of cells used [11,21]. The small number of cells used in our protocol leads to an increase in the number of replicates required to mediate the variance between and within muscle groups (Figure 2). Other variables, such as cell culture conditions, can impact myoclone expansion, from inducing faster myoclone formation to prompting cell death. For example, FGF has been shown to promote cell death at both high and low concentrations, free collagen 5 can signal to MuSCs, and the substrate can impact MuSC activation [22–24]. Finally, live-cell staining with Hoechst can hinder further use of the cells. Hoechst is cytotoxic and phototoxic to cells at high concentrations. It binds to the minor groove of the DNA and can directly interfere with replication and mitosis. Moreover, exposure to high-intensity light can cause it to release reactive oxygen species that can damage DNA. This can be mitigated by using low concentrations (1 nM is considered safe for continued cell culture) and imaging using short exposure times and small magnification objectives (4×) to acquire large fields of view [30]. These factors should be carefully considered and may need optimizing when establishing the protocol.

4. Another critical variable is the cutoff for calling myoclones. Here, we set 16 cells as the minimum number of cells required to call a myoclone. This cutoff is based on the minimum number of cell divisions we can expect the MuSCs to complete in 8 days of cell culture. A trend inversion appears between clones smaller than 16 cells or larger than 16 cells for lower hindlimb and diaphragm MuSCs, respectively (Figure 2). This small cell cutoff number also accounts for variations in activation kinetics, such as 24–48 h required for MuSCs to complete the first cell division [25,26] and variations in quiescence depth [27]. Other studies present different cutoffs and incubation periods [11,28]. It follows that the inclusion criterion set for myoclones is a critical parameter and, together with cell culture conditions and other mentioned experimental conditions, will greatly impact the binary readout of this assay.

5. The protocol results in the expansion of viable clones with minimal sample handling. In fact, cell culture medium does not need to be replaced or replenished during the experiment. If cell viability is not required for downstream experiments, the cells can be fixed and stored at 4 °C prior to microscopy analysis. We recommend analyzing the cells immediately to minimize sample handling.

6. Ensuring that the sorted single cells are MuSCs is paramount for this assay. We recommend assessing the accuracy of the FACS by measuring the purity of sorted cell populations prior to plating for the myoclone assay. This can be done either by resorting the cells or by sorting a small number and performing immunofluorescence staining for a purity marker such as Pax7 protein.

7. This assay can serve as a foundation for other assays. The MuSCs are stained with a live-cell dye and maintained in appropriate culture conditions during fluorescence imaging (i.e., warm myoclone medium), preserving the viability of the cells. It is therefore possible to perform further assays on the myoclones, such as continued cell culture, immunostaining, or RNA staining to characterize the formed myoclones [11,29]. In conclusion, we present a straightforward, versatile protocol that allows measuring stem cells’ clonogenic growth, provides insights on MuSC activation, and facilitates the study of other MuSC functions (e.g., differentiation) by isolating 60 cells per sample in total, instead of lengthy and expensive cell sorting and cell culture procedures.

## References

[1] Beumer J. and Clevers H. (2024). Hallmarks of stemness in mammalian tissues. Cell Stem Cell. 31(1): 7 24 24. 10.1016/j.stem .2023.12.006 PMC10769195 38181752

[2] de Morree A. and Rando T. A. (2023). Regulation of adult stem cell quiescence and its functions in the maintenance of tissue integrity. Nat Rev Mol Cell Biol. 24(5): 334 354 354. 10.1038/s41580-022-00568-6 PMC10725182 36922629

[3] Sambasivan R. and Tajbakhsh S. (2014). Adult Skeletal Muscle Stem Cells. Results Probl Cell Differ. 56: 191 213 213. 10.1007/978-3-662-44608-9_9 25344672

[4] Dumont N. A., Bentzinger C. F., Sincennes M. and Rudnicki M. A. (2015). Satellite Cells and Skeletal Muscle Regeneration. Compr Physiol. 5(3): 1027 1059 1059. 10.1002/j.2040-4603 .2015.tb00646.x 26140708

[5] Collins C. A., Olsen I., Zammit P. S., Heslop L., Petrie A., Partridge T. A. and Morgan J. E. (2005). Stem Cell Function, Self-Renewal, and Behavioral Heterogeneity of Cells from the Adult Muscle Satellite Cell Niche. Cell. 122(2): 289 301 301. 10.1016/j.cell .2005.05.010 16051152

[6] de Morree A., Klein J. D. D., Gan Q., Farup J., Urtasun A., Kanugovi A., Bilen B., van Velthoven C. T. J., Quarta M., Rando T. A., .(2019). Alternative polyadenylation of Pax3 controls muscle stem cell fate and muscle function. Science. 366(6466): 734 738 738. 10.1126/science.aax1694 PMC7046176 31699935

[7] Frimand Z., Das Barman S., Kjær T. R., Porpiglia E. and de Morrée A. (2022). Isolation of Quiescent Stem Cell Populations from Individual Skeletal Muscles. J Visualized Exp. e3791/64557. 10.3791/64557 36571398

[8] Hope K. and Bhatia M. (2011). Clonal interrogation of stem cells. Nat Methods. 8: S36–S40. https://doi.org/10.1038/nmeth.1590 21451511

[9] Weinstock J. S., Gopakumar J., Burugula B. B., Uddin M. M., Jahn N., Belk J. A., Bouzid H., Daniel B., Miao Z., Ly N., .(2023). Aberrant activation of TCL1A promotes stem cell expansion in clonal haematopoiesis. Nature. 616(7958): 755 763 763. 10.1038/s41586-023-05806-1 PMC10360040 37046083

[10] Mahla R. S. (2016). Stem Cells Applications in Regenerative Medicine and Disease Therapeutics. Int J Cell Biol. 2016: 1 24 24. 10.1155/2016/6940283 PMC4969512 27516776

[11] Kang J., Kanugovi A., Stella M. P. J., Frimand Z., Farup J., Urtasun A., Liu S., Clausen A. S., Ishak H., Bui S., .(2025). In vivo self-renewal and expansion of quiescent stem cells from a non-human primate. Nat Commun. 16(1): 5370 10.1038/s41467-025-58897-x PMC12187938 40555729

[12] de Morrée A., van Velthoven C. T. J., Gan Q., Salvi J. S., Klein J. D. D., Akimenko I., Quarta M., Biressi S. and Rando T. A. (2017). Staufen1 inhibits MyoD translation to actively maintain muscle stem cell quiescence. Proc Natl Acad Sci USA. 114(43): e1708725114. https://doi.org/10.1073/pnas.1708725114 PMC5664522 29073096

[13] Baghdadi M. B., Castel D., Machado L., Fukada S. I., Birk D. E., Relaix F., Tajbakhsh S. and Mourikis P. (2018). Reciprocal signalling by Notch–Collagen V–CALCR retains muscle stem cells in their niche. Nature. 557(7707): 714 718 718. 10.1038/s41586-018-0144-9 PMC5985950 29795344

[14] Cecchini A., Loreti M., Kaufman C. D., Stamenkovic C., Guarnaccia G., Renero A., Nicoletti C., Kervadec A., Mayer D., Delaware S., .(2025). Tenascin-C from the tissue microenvironment promotes muscle stem cell maintenance and function through Annexin A2. Commun Biol. 8(1): 1709 10.1038/s42003-025-09189-z PMC12680774 41350439

[15] Gromova A., Tierney M. and Sacco A. (2015). FACS-based Satellite Cell Isolation From Mouse Hind Limb Muscles. Bio Protoc. 5(16): e1558. 10.21769/bioprotoc.1558 PMC5034768 27668269

[16] Liu L., Cheung T. H., Charville G. W. and Rando T. A. (2015). Isolation of skeletal muscle stem cells by fluorescence-activated cell sorting. Nat Protoc. 10(10): 1612 1624 1624. 10.1038/nprot.2015 .110 PMC4793971 26401916

[17] Jensen J. B., Møller A. B., Just J., Mose M., de Paoli F. V., Billeskov T. B., Fred R. G., Pers T. H., Pedersen S. B., Petersen K. K., .(2021). Isolation and characterization of muscle stem cells, fibro-adipogenic progenitors, and macrophages from human skeletal muscle biopsies. Am J Physiol, Cell Physiol. 321(2): C257–C268. 10.1152/ajpcell.00127 .2021 34106790

[18] Sacco A., Doyonnas R., Kraft P., Vitorovic S. and Blau H. M. (2008). Self-renewal and expansion of single transplanted muscle stem cells. Nature. 456(7221): 502 506 506. 10.1038/nature07384 PMC2919355 18806774

[19] Pasut A., Oleynik P. and Rudnicki M. A. (2011). Isolation of Muscle Stem Cells by Fluorescence Activated Cell Sorting Cytometry. Methods Mol Biol. 798: 53 64 64. 10.1007/978-1-61779-343-1_3 22130830

[20] Barman S., Frimand Z. and Morree A. (2023). Absolute Quantification of mRNA Isoforms in Adult Stem Cells Using Microfluidic Digital PCR. Bio Protoc. 13(17): e4811. 10.21769/bioprotoc.4811 PMC10501916 37719075

[21] Wilkinson A. C., Igarashi K. J. and Nakauchi H. (2020). Haematopoietic stem cell self-renewal in vivo and ex vivo. Nat Rev Genet. 21(9): 541 554 554. 10.1038/s41576-020-0241-0 PMC7894993 32467607

[22] Rando T. A. and Blau H. M. (1994). Primary mouse myoblast purification, characterization, and transplantation for cell-mediated gene therapy. J Cell Biol. 125(6): 1275 1287 1287. 10.1083/jcb.125 .6.1275 PMC2290930 8207057

[23] Gilbert P. M., Havenstrite K. L., Magnusson K. E. G., Sacco A., Leonardi N. A., Kraft P., Nguyen N. K., Thrun S., Lutolf M. P., Blau H. M., .(2010). Substrate Elasticity Regulates Skeletal Muscle Stem Cell Self-Renewal in Culture. Science. 329(5995): 1078 1081 1081. 10.1126/science.1191035 PMC2929271 20647425

[24] Quarta M., Brett J. O., DiMarco R., De Morree A., Boutet S. C., Chacon R., Gibbons M. C., Garcia V. A., Su J., Shrager J. B., .(2016). An artificial niche preserves the quiescence of muscle stem cells and enhances their therapeutic efficacy. Nat Biotechnol. 34(7): 752 759 759. 10.1038/nbt.3576 PMC4942359 27240197

[25] Rodgers J. T., King K. Y., Brett J. O., Cromie M. J., Charville G. W., Maguire K. K., Brunson C., Mastey N., Liu L., Tsai C. R., .(2014). mTORC1 controls the adaptive transition of quiescent stem cells from G0 to GAlert. Nature. 510(7505): 393 396 396. 10.1038/nature13255 PMC4065227 24870234

[26] Siegel A. L., Kuhlmann P. K. and Cornelison D. (2011). Muscle satellite cell proliferation and association: new insights from myofiber time-lapse imaging. Skelet Muscle. 1(1): 7 10.1186/2044-5040-1-7 PMC3157006 21798086

[27] Rocheteau P., Gayraud-Morel B., Siegl-Cachedenier I., Blasco M. A. and Tajbakhsh S. (2012). A Subpopulation of Adult Skeletal Muscle Stem Cells Retains All Template DNA Strands after Cell Division. Cell. 148: 112 125 125. 10.1016/j.cell .2011.11.049 22265406

[28] Tierney M. T., Stec M. J., Rulands S., Simons B. D. and Sacco A. (2018). Muscle Stem Cells Exhibit Distinct Clonal Dynamics in Response to Tissue Repair and Homeostatic Aging. Cell Stem Cell. 22(1): 119 127 127 .e3. 10.1016/j.stem .2017.11.009 PMC5945549 29249462

[29] Shams A. S. and Kyba M. (2023). The Satellite Cell Colony Forming Cell Assay as a Tool to Measure Self-Renewal and Differentiation Potential. Methods Mol Biol. 2640: 45 55 55. 10.1007/978-1-0716-3036-5_4 36995586

[30] Fuchs H., Jahn K., Hu X., Meister R., Binter M. and Framme C. (2023). Breaking a Dogma: High‐Throughput Live‐Cell Imaging in Real‐Time with Hoechst 33342. Adv Healthcare Mater. 12(20): e202300230. https://doi.org/10.1002/adhm.202300230 PMC11468280 36934382

